# The role of internet integration in relation to farmers’ income and perceived social fairness

**DOI:** 10.1371/journal.pone.0337067

**Published:** 2025-12-29

**Authors:** Ke Zheng, Yanan Feng, Ruyu Zhang, Yufeng Li, Xiumin Wu

**Affiliations:** College of Management, Sichuan Agricultural University, Chengdu, Sichuan, China; Xi'an Jiaotong-Liverpool University, CHINA

## Abstract

This study investigates how Internet embeddedness moderates the relationship between farmers’ income and perceived social fairness in rural China through the integrated lens of social comparison and relative deprivation theories. Analyzing 1,036 rural households in Sichuan Province via Oprobit regression with instrumental variables, we find that while absolute income growth positively affects PSF, Internet usage significantly weakens this relationship through three mechanisms: (1) expanded reference groups that facilitate upward social comparisons, (2) algorithmic distortion of economic reality through selective content exposure, and (3) cognitive overload from excessive income-related information. These findings reconcile conflicting evidence in the income-fairness literature by demonstrating how digital connectivity transforms traditional comparison processes. This study contributes to development economics by highlighting the dual role of technology in rural welfare, while enabling information access, it may inadvertently undermine fairness perceptions through psychological and algorithmic channels. The policy implications suggest the need to complement income growth strategies with digital literacy programs and platform governance to mitigate comparison biases.

## Introduction

Perceived social fairness (PSF), a core construct in social mentality research [1,2], represents not only individuals’ subjective judgments about distributive justice [3] but also serves as a crucial predictor of collective action and policy compliance, which fundamentally shape national development and social stability [4–6]. In the contemporary context of the global imbalance intertwined with the digital revolution, farmers’ PSF exhibits unique complexity. On the one hand, China has witnessed 11 consecutive years of absolute income growth in rural areas. Official statistics from the National Bureau of Statistics reveal that the annual income of rural residents increased from 7,919 yuan in 2012–21,691 yuan in 2023 [7]. Such remarkable income growth would theoretically enhance farmers’ social well-being [8]. On the other hand, official statistics from the National Bureau of Statistics estimates indicate that China’s Gini coefficient remained in the range of 0.46--0.47 in 2022, significantly exceeding the global average of 0.44. Notably, the urban‒rural income gap constitutes one of the primary drivers of fluctuations in China’s Gini coefficient [9]. Drawing upon social comparison theory, the relationship between income and PSF is nonlinear. PSF derives not only from absolute improvements in material living standards but also from social comparisons of income [10,11]. This dynamic has been fundamentally transformed by China’s rural Internet penetration rate, reaching 67.4% [12], which has effectively dissolved the geographical constraints on farmers’ reference groups [13].

In the current digital era, pervasive Internet embeddedness has fundamentally transformed farmers’ information accessibility and social networks [14,15], thereby introducing unprecedented complexity to the income‒fairness relationship. Farmers’ PSF transcends a simple linear function of income level, instead constituting a sophisticated outcome embedded in a tripartite transformation process that integrates economic dimensions, technological mediation and psychological computation [16–18].

From an economic perspective, the complexity of the impact of income manifests through both absolute and relative income effects. The absolute income hypothesis emphasizes the role of income growth. Drawing on Sen’s (1999) capability approach, we can identify two distinct pathways through which income affects PSF: (1) the enabling pathway: income growth → increased investment in healthcare/education → expanded capabilities → enhanced fairness perception; and (2) the blocked pathway: when institutional voids exist (e.g., lack of access to quality public services), income growth fails to translate into substantive freedoms, resulting in diminishing marginal returns to fairness perceptions [16]. In comparison, relative income has also received widespread scholarly attention. A growing body of research suggests that the increase in absolute income may be less significant than relative income is, and importantly, an increase in absolute income does not necessarily translate to an improvement in relative income [5,19,20].

The advancement of digital technologies and widespread Internet adoption have significantly expanded the comparative scope of relative income perception. This technological shift has transformed the fairness perception effect of income growth by extending reference groups from geographically constrained local samples to unrestricted national and even global samples [21,22]. Such expansion fundamentally alters the association pattern between economic factors and psychological outcomes. Moreover, this impact is further complicated by the dual effects inherent in Internet embeddedness itself.

The dual effects of Internet adoption have been empirically validated across multiple dimensions. In terms of positive impacts, the Internet enhances individuals’ capabilities—including social participation, self-expression, information access, and network building—thereby strengthening their agency over life circumstances and ultimately advancing human development [23–25]. Specifically, it offers a broad platform for entertainment [26], communication [27], and shopping, which can reduce depression [28], enhance subjective well-being [29], and improve PSF [22]. In terms of negative effects, the Internet has fundamentally disrupted the traditional insularity of rural societies by enabling farmers to directly observe income disparities and lifestyle differences across urban‒rural divides and social strata [30,31]. This emerging cross-stratum comparison dynamic significantly intensifies feelings of relative deprivation [32–34]. More critically, algorithmic recommendation technologies may confine farmers to particularized information ecosystems, thereby amplifying preexisting cognitive biases through filter bubble or information cocoon effects [35–37]. Furthermore, the Internet’s systemic preference for disseminating negative content may progressively erode farmers’ institutional and societal trust [38,39], creating a self-reinforcing cycle of skepticism and disengagement.

In summary, this study focuses on a pivotal negative externality of Internet penetration: its capacity to expand reference groups for income comparisons. This expansion may lead individuals to perceive inadequate relative income growth or unmet expectations, thereby intensifying feelings of relative deprivation. Consequently, the positive impact of absolute income growth on farmers’ PSF could be substantially attenuated. Stated formally, our central research question examines the following: Does Internet-induced transformation of reference systems weaken the positive association between absolute income and farmers’ PSF?

Research has rarely explored the complex role of Internet embedding in the pathway through which income affects PSF, particularly from the perspectives of social comparison and relative deprivation theories. Given the increasing role of the Internet in farmers’ daily lives, it is crucial to examine how Internet embedding influences the relationship between income and PSF.

## Hypotheses

### Income and PSF

The interplay between income dynamics and farmers’ PSF can be understood through the lens of social comparison theory and its extension, relative deprivation theory. These frameworks highlight how individuals evaluate their socioeconomic status by comparing themselves to reference groups, shaping their emotional responses to fairness and justice [40]. Central to this process is the selection of reference objects, which typically fall into two categories: (1) horizontal comparisons with peers and (2) vertical comparisons with one’s past [41]. The choice of reference is not arbitrary; it depends on the accessibility of information about others’ incomes or historical data [42,43]. When individuals perceive their income as meeting expectations, they experience positive emotions and a sense of fairness. Conversely, disadvantageous comparisons may trigger relative deprivation, undermining trust in societal institutions and distorting perceptions of educational access or social mobility [44,45].

Currently, the income level of farmers has steadily increased compared with that in the past. On the one hand, China has implemented the “Targeted Poverty Alleviation” campaign in rural areas, aiming to eliminate absolute poverty while ameliorating relative poverty, which has significantly improved rural poverty conditions [44,45]; on the other hand, agricultural incomes have risen with the modernization and enhancement of traditional agricultural practices through the adoption of green production techniques and advanced agricultural operations [46,47]. Moreover, non-agricultural incomes have also grown, partly due to the establishment of manufacturing bases and the development of secondary and tertiary industries, such as rural tourism, in rural areas. Another contributing factor is the increased employment of farmers in urban areas, leading to higher non-agricultural incomes [48].

From the perspective of absolute income’s impact on PSF, income growth directly enhances farmers’ sense of social equity through economic capacity expansion. Sen’s (1999) capability approach theoretically underscores that economic resources serve as fundamental conditions for achieving substantive freedom, which specifically manifests in enhanced consumption capacity, increased productive investment and improved risk resilience [49,50]. Moreover, when income growth involves both extensive coverage and temporal sustainability, it reduces economic uncertainty while increasing expectations, thereby significantly strengthening farmers’ perceived substantive gains [51,52]. Notably, Sen’s capability approach places greater emphasis on the critical transition from income growth to the expansion of substantive individual capabilities, a process fundamentally contingent on institutional safeguards [16]. Under China’s ongoing rural revitalization strategy, the translation of farmers’ income growth into expanded substantive capabilities is facilitated through substantial government investments and targeted institutional arrangements, including improved rural healthcare accessibility, enhanced educational opportunities, upgraded housing policies, and inclusive financial initiatives [18,53]. Concurrently, rising incomes coupled with the nationwide implementation of digital village construction have enabled farmers to actively participate in and benefit extensively from digital empowerment effects, leading to widespread advancements in personal development [23–25]. The rise in income has improved the quality of life of farmers, allowing them to perceive their lives as better than before. As a result, they are less likely to experience feelings of deprivation and are likely to perceive greater PSF due to the increase in absolute income.

From the perspectives of social comparison theory and relative deprivation theory, farmers in traditional rural societies demonstrate a greater inclination toward vertical income comparisons [18]. This is attributed to the communal nature of traditional rural society, where information is predominantly exchanged within close-knit local communities. Farmers usually receive information from their neighbors or traditional media channels [54]. The range of reference objects is relatively narrow and is usually limited to local villages or nearby towns. The difference between the income of these individuals and that of other locals is not substantial [55]. They are more predisposed to longitudinal comparisons, contrasting with their own past. This aligns with earlier research by Van Praag, Kapteyn and Van Herwaarden suggesting that individuals in less urbanized areas prioritize their own income over that of others [56].

Based on the above discussion, we propose the following hypothesis:

Hypothesis 1: An increase in farmers’ income positively affects their PSF.

### The moderating role of internet embedding

Internet embeddedness provides farmers with transformative capabilities that may directly enhance socioeconomic well-being. Empirical evidence demonstrates its multidimensional benefits: (1) Economic Agency: Increased income through e-commerce [57] and non-agricultural employment [58]; (2) Production Modernization: Improved efficiency [59] and green technology adoption [60]; (3) Social Capital: Enhanced community engagement [61] and reduced isolation [62,63].

However, the paradox is that empowerment does not equal an enhanced PSF. Despite those benefits, Internet use simultaneously undermines PSF through (1) horizontal comparisons and upward comparison traps: farmers increasingly compare themselves with national and urban/elite reference groups [22]; (2) algorithmic distortion: platforms may amplify visible inequality [64–67]; and (3) cognitive overload: information abundance exacerbates awareness of unattainable lifestyles [68]. Crucially, these mechanisms disproportionately affect psychological assessments of equity, even as objective conditions improve.

#### Horizontal comparisons and upward comparison traps.

Internet embeddedness may reshape farmers’ social comparison patterns through two critical dimensions, thereby fundamentally altering their PSF. First, it dramatically expands the informational basis for comparisons by enabling access to income-related data previously unavailable in traditional rural social contexts [22]. This qualitative transformation in information accessibility significantly intensifies both the frequency and scope of comparisons, with a particular emphasis on enhancing relative income awareness. In traditional rural societies, farmers typically have lower incomes and limited access to information from others. However, with the advent of Internet embedding, farmers’ social interactions with weak ties increase, their access to information broadens, and they are more likely to learn about others’ incomes. Second, Internet embeddedness alters the directional characteristics of social comparisons. While classical theories identify three comparison directions—upward, lateral, and downward [33,40]—platform algorithms and content distribution mechanisms systematically amplify the propensity for upward comparisons [21,22,66]This systematic skew in comparison directionality leads farmers to more frequently contrast their circumstances with more advantaged reference groups, which could lead to a lower PSF [22].

#### Algorithm distortion.

Algorithmic distortion refers to the systematic biases introduced by digital platforms’ content curation systems that disproportionately amplify certain types of information while suppressing others [64,65]. In rural contexts, this may be manifested through visibility bias caused by platforms, where platforms prioritize emotionally charged and engagement-driven content—including displays of extreme wealth or poverty [66,69]—to maximize virality. Nonrepresentative cases (e.g., “Taobao village millionaires”) receive disproportionate visibility, whereas urban middle-class consumption patterns dominate feeds because of their higher engagement potential. This dynamic exacerbates farmers’ information cocoon effects [35,36]. Through both passive exposure and active user engagement, farmer users become disproportionately exposed to curated high-income lifestyle content, which systematically fosters a “pervasive affluence” cognitive bias by leading them to misperceive platform-amplified extreme cases as representative societal norms. This distorted reality perception subsequently magnifies dissatisfaction with personal economic standing while heightening perceptions of distributive injustice in resource allocation.

#### Cognitive overload.

Cognitive overload refers to a state of psychological stress caused by information input exceeding an individual’s cognitive processing capacity, representing a fundamental imbalance between information intake and cognitive resources [68]. In the Internet era, individuals’ daily exposure to digital information has increased exponentially compared with that of traditional societies [14], resulting in increasingly fragmented attention [70] and continuous information evaluation that depletes mental energy while impairing rational judgment [71]. For farmers, this information deluge creates sustained exposure to lifestyles far beyond their economic means—from luxury consumption patterns to elite career trajectories—making individuals more acutely aware of the gap between “ideal lifestyles” and “actual circumstances.” This cognitive state not only fuels materialistic aspirations but also leads individuals to attribute unattainable living standards to unequal social opportunities, thereby diminishing their evaluations of PSF [72].

Therefore, we propose the following hypothesis:

Hypothesis 2: Internet embeddedness plays a negative moderating role between income and PFS.

**Fig 1:**
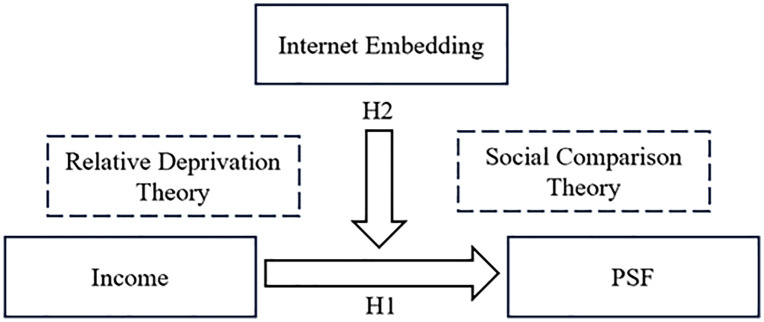
Theoretical framework.

The theoretical model of income, PSF, and Internet embeddedness constructed in this paper on the basis of social comparison theory and relative deprivation theory is shown in Fig 1.

## Methodology

### Model

Since this paper investigates the effect of income on the PSF, the dependent variable, the sense of PSF, is an ordinal variable given that it takes values ranging from 1--5. To enhance the robustness of the empirical research in this paper, the Oprobit method is used for estimation. The model is set up as follows:


$$Y_{i}=F({\text{a}}_{1}x_{1i}+{\text{a}}_{2}x_{2i}+{\text{a}}_{3}x_{1i}x_{2i}+\theta_{0}X_{0}+\mu_{i})$$ (1)

In equation (1), $Y_{i}$ represents farmer $i^{\prime }$’s perceptions of social fairness, $x_{1i}$ represents the core explanatory variable income of farmer i, $x_{2i}$ represents the moderating variable Internet embedding of farmer i, $X_{0}$ represents the control variable, and $\mu_{i}$ represents the random error term.


$$F(Y_{i}^{*})=\left\{ \begin{array}{c} \;\;1,{\;\;\;\;\;\;\;\;\;Y}_{i}^{*}<=r_{1}\\ 2,\;r_{1}<Y_{i}^{*}<=r_{2}\\ 3,\;r_{2}<Y_{i}^{*}<=r_{3}\\ 4,\;r_{3}<Y_{i}^{*}<=r_{4}\\ \;5,\;r_{4}<Y_{i}^{*}\;\;\;\;\;\;\;\;\;\;\;\; \end{array}\right.$$ (2)

In equation (2), $r_{1}<\;r_{2}<\;r_{3}<r_{4}$ represents the thresholds, which are all parameters to be estimated. When ${\;Y}_{i}^{*}$ is below the critical value $r_{1}$, farmer i decides “Resolutely believe in social unfairness”; when $Y_{i}^{*}$ is between $r_{1}$ and $r_{2}$, farmer i decides “Generally, believed that society is unfair”; and when ${\;Y}_{i}^{*}$ exceeds $r_{4}$, farmer i will “Resolutely believe in social fairness”.

$Y_{i}^{*}$ is the unobservable continuous variable, which can be viewed as the latent variable for farmer i’s PFS. Its estimation equation is as follows:


$$Y_{i}^{*}={\text{a}}_{1}x_{1i}+{\text{a}}_{2}x_{2i}+{\text{a}}_{3}x_{1i}x_{2i}+\theta_{0}X_{0}+\mu_{i}$$ (3)

The variables in the equation are consistent with the definitions mentioned earlier.

### Sample sources and variable descriptions

#### Sample sources.

To test the core hypotheses outlined in the theoretical analysis, this study selected Sichuan Province as the research area. Sichuan, a major agricultural province in China, is known for its geographic, ecological, and ethnic diversity. According to the 2019 Statistical Yearbook of Urban and Rural Construction in China, Sichuan has the largest number of natural villages in the country, making it a focal area for rural revitalization and digital village initiatives. In 2014, the Chinese government launched the “National E-Commerce Demonstration County” program to promote rural industry development, stimulate employment and entrepreneurship, and increase farmers’ income and wealth. These demonstration counties, which exemplify advanced rural e-commerce development, have been instrumental in increasing rural incomes [73]. Sichuan now hosts the highest number of comprehensive e-commerce demonstration counties nationwide and has established over 10,000 village-level e-commerce logistics points, significantly boosting farmers’ income levels. Prior research has indicated that e-commerce demonstration county development positively impacts farmers’ income [74]. Focusing on these counties provides a suitable context for examining the role of Internet embedding in the relationship between income and perceived fairness. The sample also includes e-commerce demonstration counties in Sichuan’s high-altitude areas, where rural revitalization has spurred rapid tourism industry growth, which is now a key economic pillar [75]. As a result, income levels in these high-altitude regions have substantially increased, adding to the representativeness of our sample.

Data collection employed a combination of typical, stratified, and random sampling methods. From Sichuan Province’s 181 county-level units, e-commerce demonstration counties shortlisted between 2014 and 2021 were selected as the sample pool, totaling 105 counties identified through public information sources. On the basis of the “2020 China County Digital Rural Index,” released by the Institute of New Rural Development at Peking University and the Alibaba Research Institute in 2022, the sample pool was divided into three categories of county digital empowerment index zones: high, medium, and low. Within these three index levels, 46 villages across 38 e-commerce demonstration counties in 14 cities/states were selected as sample villages. Field surveys conducted in these villages yielded 1,036 valid questionnaires.

Our research does not involve minors, and all participants have provided informed consent. Each field investigation team consists of at least two interviewers. One interviewer seeks the respondent’s consent for data collection, analysis, and publication, explaining the study’s purpose, how their data will be used, and any potential risks. The second interviewer witnesses the explicit consent process. We ensure that this research is conducted under the supervision of our university and with the subjects’ consent. We are committed to safeguarding the participants’ data and will not disclose or release personal information inappropriately. The data has been anonymized, and this study poses no risk to human subjects.

#### Descriptive statistics and variable selection

***Independent variable.*** Income. The annual income level of the respondent in the past 12 months. There are 10 levels ranging from “less than 10 thousand yuan” to “more than 2 million yuan”, which are measured by asking “In the past 12 months, in which of the following ranges did your household income fall”.

***Dependent variable.*** PSF. An individual’s perception of whether society is fair. There are multiple dimensions of PSF, but it is the overall PSF that drives people’s behavior [76]. Hence, this paper measures PSF through respondents’ overall judgment and comprehensive evaluation of social fairness through the item “I think society is fair now.” A five-point Likert scale was used, with scores ranging from 1 “strongly disagree” to 5 “strongly agree”. The higher the score is, the greater the individual’s PSF.

***Moderator variable.*** Internet embedding. The degree to which an individual accesses information from the Internet. Currently, the Internet is a common way to access online information, but access to the Internet presupposes the use of electronic devices. Some previous studies have investigated individuals’ Internet use by asking “whether you have installed a device that can access the Internet or broadband” [77] and “whether you use a mobile device to access the Internet” [73]. After confirming that respondents could access the Internet, some studies delved deeper into individual Internet usage in terms of the frequency, extent, scope, and type of Internet use [78–80]. Considering that the embeddedness of the Internet in farmers’ lives mainly involves social interaction and that the provision of and access to the corresponding information is basically obtained through social interaction, we chose indicators related to social information acquisition to measure individuals’ Internet embedding.

Specifically, this study measures four items, namely, “the number of social groups joined on WeChat, QQ, or other platforms with over 50 members in the past year,” “the number of WeChat friends,” “methods of making friends online,” and “the number of online friends made in the past year”. According to the actual choices of the respondents, factor analysis was used for dimensionality reduction; those below the mean were assigned a value of 0, and those above the mean were assigned a value of 1.

Table 1 presents the results of the KMO and Bartlett tests conducted on the selected items prior to factor analysis dimensionality reduction. The results indicate that KMO = 0.738 (greater than 0.6), and the chi-square value for the Bartlett test is 1004.206, which is highly significant. This suggests that the assumption of independence among the selected items does not hold, demonstrating a satisfactory level of data intercorrelation in the questionnaire and suitability for factor analysis. The factor loadings and entropy weights can be found in Table 1.

**Table 1:** Factor Dimension Reduction and Entropy Weighting of Internet Embedding.

Variables	Items	Factor Loading/ entropy weight	KMO	Bartlett test
**Internet Embedding**	the number of social groups joined on WeChat, QQ, or other platforms with over 50 members in the past year	0.786	0.738	1004.206***
	the number of WeChat friends	0.716		
	the number of online friends made in the past year	0.673		
	methods of making friends online	0.848		

Note: *** p < 0.01, ** p < 0.05, * p < 0.1.

***Control variables.*** These variables include as many variables as possible that may affect the sense of fairness, including demographic variables such as “gender, age, marital status, risk-taking, education”; social variables such as length of time working outside, length of residence in rural areas, income diversity, and agricultural production diversity; and geographic variables such as “distance to town,” totaling nine variables. In addition, regional variables are also controlled.

After the questionnaires were collected and processed in series, we collected 1036 valid questionnaires. Specific measures and descriptive statistics for the independent, dependent, moderating, and control variables are presented in Table 2.

**Table 2:** Measurement Explanation of Variables and Descriptive Statistics.

Variables	Concept Connotation	Metrics	Obs.	Mean	SD	Min	Median	Max
**Dependent Variable**								
PSF	Perceived social fairness	Measured by the question “I think society is fair now”.	1036	3.423	1.071	1.000	4.000	5.000
**Independent Variable**								
Income	Respondents’ annual income in the last 12 months	There are 10 levels ranging from “less than 10 thousand yuan” to “more than 2 million yuan”,	1036	3.635	1.451	1.000	4.000	11.000
**moderator**								
Internet embedding	The degree to which an individual accesses information from the Internet	A total of four question items, such as the number of online friends made in the past year, were downgraded using factor analysis; above average = 1, below average = 0	1036	0.458	0.498	0.000	0.000	1.000
**Control variables**								
Gender	Gender of respondents	0 = male; 1 = Female	1036	0.462	0.499	0.000	0.000	1.000
Age	Age of respondents	8 age brackets in descending order from 0 to 76 years of age	1036	3.968	1.509	1.000	4.000	8.000
Marital Status	Whether the respondent is in a marriage	0= in a nonmarital status; 1 = in a marital status	1036	0.624	0.485	0.000	1.000	1.000
Risk-Taking	Respondents’ preference for project investment	1 = Low risk-low return; 2 = Medium risk-medium return;3 = High risk-high return	1036	0.586	0.610	1.000	2.000	3.000
Education	Respondents’ level of education	There are a total of 8 levels from never attending to obtaining a PhD degree, with values ranging from 0 to 7 in sequence	1036	2.389	1.482	0.000	2.000	7.000
Length of outside working time	total time spent working outside the home	Divided into six levels from 0 to 10 years	1036	3.437	1.941	1.000	3.000	6.000
Residence time in rural area	Length of time spent living in the countryside during the year	From “not residing” to “more than half a year” are assigned values from 1 to 5.	1036	3.986	1.199	1.000	4.000	5.000
Diversity of income	Types of household income sources	There are 10 levels according to the types of occupations practiced, including a dozen types of occupations such as “farmer”.	1036	1.295	0.695	0.000	1.000	5.000
Agricultural production	Whether engaged in agricultural production	0 = engaged in non-agricultural related activities; ;1 = Engaged in agriculture-related activities	1036	0.411	0.492	0.000	0.000	1.000
Distance to town	Distance to the nearest town	——	1036	41.151	58.261	1.000	18.000	260.000

## Results and discussion

The correlations of all the variables are presented in Table 3. With a mean variance inflation factor (VIF) value of 1.56, which is less than 2, multicollinearity is not a concern. The Oprobit method was employed to test the hypotheses, wherein the control, explanatory, and moderating variables were included in the regression equations for the three models. The instrumental variables approach was subsequently utilized to address endogeneity, and a robustness test was subsequently conducted through replacement regression models. Finally, we individually examined the heterogeneity of the dimensions “Length of outside working time” and “Residence time in rural areas”.

**Table 3:** Correlation analysis.

VARIABLES	Income	PSF	Gender	Age	Marital status	Risk-Taking	Education	Length of outside working outside	Residence time in rural area	Diversity of income	Agricultural production	Distance to town
**Income**	1											
**PSF**	0.137***	1										
**Gender**	−0.199***	−0.106***	1									
**Age**	−0.0330	0.00800	−0.143***	1								
**Marital status**	0.0380	0.0150	0.0130	0.381***	1							
**Risk-Taking**	0.206***	0.067**	−0.091***	−0.301***	−0.201***	1						
**Education**	0.240***	−0.0500	0.092***	−0.523***	−0.193***	−0.027	1					
**Length of outside working time**	0.151***	−0.0420	−0.219***	0.334***	0.210***	0.0260	−0.116***	1				
**Residence time in rural area**	−0.103***	0.078**	0.0260	0.353***	0.182***	−0.243***	−0.322***	−0.079**	1			
**Diversity of income**	0.174***	0.145***	−0.118***	0.0380	0.0350	0.111***	−0.054*	0.123***	0.055*	1		
**Agricultural production**	−0.059*	0.126***	−0.0120	0.257***	0.135***	−0.108***	−0.312***	−0.074**	0.344***	0.481***	1	
Distance to town	0.093***	0.254***	−0.130***	−0.078**	−0.0380	−0.053*	−0.178***	−0.087***	0.056*	0.204***	0.271***	1

Note: *** p < 0.01, ** p < 0.05, * p < 0.1.

### Hypothesis testing

#### Factors influencing farmers’ PSF.

Table 4 Equation (1) displays the results with only control variables entered into the regression. The results indicate that risk-taking, length of residence in a rural area, distance from the county, and some of the regional variables significantly affect an individual’s PSF.

**Table 4:** Oprobit regression results.

VARIABLES	Equation 1	Equation 2	Equation 3
	PSF	PSF	PSF
**Income**		0.096***	0.138***
		(3.25)	(3.84)
**Internet embedding**			0.517**
			(2.43)
**Income##Internet embedding**			−0.102*
			(−1.91)
**Gender**	−0.101	−0.065	−0.063
	(−1.40)	(−0.88)	(−0.85)
**Age**	0.031	0.027	0.037
	(0.97)	(0.85)	(1.16)
**Marital Status**	0.086	0.068	0.074
	(1.06)	(0.84)	(0.92)
**Risk-Taking**	0.188***	0.155**	0.162**
	(2.92)	(2.41)	(2.47)
	0.045	0.027	0.005
**Education**	(1.48)	(0.85)	(0.14)
**Length of outside working time**	0.003	0.000	−0.002
	(0.13)	(0.02)	(−0.09)
**Residence time in rural area**	0.068**	0.070**	0.067*
	(1.98)	(2.03)	(1.94)
**Diversity of income**	0.054	0.013	0.003
	(0.91)	(0.21)	(0.05)
**Agricultural production**	0.010	0.042	0.043
	(0.12)	(0.49)	(0.50)
**Distance to town**	0.005***	0.005***	0.004***
	(7.46)	(6.44)	(6.03)
**Observations**	1,036	1,036	1,036
**City**	Control	Control	Control

Note: The parentheses show robust z statistics. *** p < 0.01, ** p < 0.05, * p < 0.1.

Risk-taking was significant at the 1% level of significance for farmers’ sense of social justice, indicating that the more risk-averse and high-return people were, the more they felt socially fair. One explanation is that more adventurous people may be more likely to feel that inputs are proportional to outputs and that they may be less likely to make risky investments if they feel social fairness.

The length of residence in rural areas in a year also influences farmers’ PSF. The results of the study indicated that the longer the duration of residence in rural areas is one year, the greater the farmers’ PSF. This finding is similar to that of Knight, Lina and Gunatilaka (2009) [18]. The longer individuals reside in rural areas, the more likely they are to perceive that living conditions in their place of residence are improving, leading to an increase in personal well-being and a greater probability of perceiving society as becoming fairer.

In addition, the greater the distance of an individual’s residence from the county seat is, the greater the PSF. The greater the distance of an individual’s residence from the county seat is, the stronger the perception of fairness in rural areas. The reasons may be the occlusion of information and the fact that they feel little deprivation when there is little difference between the individuals in the environment in which they live.

However, sex, age, marital status and education level were not significant. This is not in line with some of the previous studies [81]. As far as gender is concerned, the probable reason for this is that society currently gives roughly equal rights and opportunities to men and women; hence, the effect of gender may be insignificant. In terms of age and education, the sample population selected for this study is a group of farmers whose age is concentrated in the middle-aged group and who generally have a low level of educational attainment; thus, the regression results may be insignificant. In addition, individuals are mostly free to marry, so marriage may not directly affect the individual’s evaluation of the PSF.

#### The impact of income on PSF.

Table 4 Equation 2 displays the regression results for H1, showing that income positively affects farmers’ PSF at the 1% significance level. This result remains consistent even with the inclusion of the regional dummy variable, reinforcing H1. Some previous studies have confirmed the positive relationship between income and individual well-being [82,83]. According to the logic of economics, the well-being of an individual is related to absolute income [84]. Overall, the higher an individual’s income is, the more he or she tends to be in a socially advantaged position. Individuals in socially advantaged positions are less likely to feel deprived. Hence, an increase in absolute income significantly affects an individual’s sense of PSF.

#### Moderating effects of internet embedding.

Table 4 Equation 3 presents the regression results for the moderating effect, showing that Internet embedding negatively moderates the effect of income on PSF at the 10% significance level. These results support evidence from previous observations [22]. Internet embedding can increase the PSF because the increase in absolute income is suppressed by expanding the range of farmers’ income comparison, validating H2. The Internet, as a rapidly developing new information dissemination channel, has become a key means of accessing information. The Internet has lowered the threshold of information dissemination, which inevitably contains more negative news for farmers [85]. The existence of such news may induce individuals to have a lower sense of PSF. In addition, the Internet has increased the material desires of individuals while expanding the income reference range [72]. When the economic level of the reference object is better, the individual is more likely to trigger the individual to develop a sense of relative deprivation [86]. The creation of a sense of relative deprivation induces the individual to feel increasingly PSF.

Table 5 displays the average marginal effects of the core explanatory variable income on farmers’ sense of PSF. The results indicate that for each unit increase in income, the likelihood of farmers perceiving social unfairness decreases by 1.3%, whereas the probability of PSF increases by 2.8%. The regression results are consistent with the hypotheses, further confirming the robustness of the findings.

**Table 5:** Marginal Effect of Income.

	Marginal effect				
VARIABLES	PSF = 1	PSF = 2	PSF = 3	PSF = 4	PSF = 5
	(1)	(2)	(3)	(4)	(5)
**Income**	−0.013***	−0.022***	−0.015***	0.021***	0.028***
	(0.003)	(0.006)	(0.004)	(0.006)	(0.008)
**Internet embedding**	−0.047**	−0.083**	−0.056**	0.080**	0.106**
	(0.020)	(0.034)	(0.024)	(0.033)	(0.044)
**Income##Internet embedding**	0.009*	0.016*	0.011*	−0.016*	−0.021*
	(0.005)	(0.009)	(0.006)	(0.008)	(0.011)
**Gender**	0.006	0.010	0.007	−0.010	−0.013
	(0.007)	(0.012)	(0.008)	(0.011)	(0.015)
**Age**	−0.003	−0.006	−0.004	0.006	0.008
	(0.003)	(0.005)	(0.003)	(0.005)	(0.006)
**Marital Status**	−0.007	−0.012	−0.008	0.012	0.015
	(0.007)	(0.013)	(0.009)	(0.013)	(0.017)
**Risk-Taking**	−0.015**	−0.026**	−0.018**	0.025**	0.033**
	(0.006)	(0.011)	(0.007)	(0.010)	(0.013)
**Education**	−0.000	−0.001	−0.000	0.001	0.001
	(0.003)	(0.005)	(0.003)	(0.005)	(0.007)
**Length of outside working time**	0.000	0.000	0.000	−0.000	−0.000
	(0.002)	(0.003)	(0.002)	(0.003)	(0.004)
**Residence time in rural area**	−0.006*	−0.011*	−0.007*	0.010*	0.014*
	(0.003)	(0.006)	(0.004)	(0.005)	(0.007)
**Diversity of income**	−0.000	−0.000	−0.000	0.000	0.001
	(0.006)	(0.010)	(0.007)	(0.010)	(0.013)
**Agricultural production**	−0.004	−0.007	−0.005	0.007	0.009
	(0.008)	(0.014)	(0.009)	(0.013)	(0.018)
**Distance to town**	−0.000***	−0.001***	−0.000***	0.001***	0.001***
	(0.000)	(0.000)	(0.000)	(0.000)	(0.000)
**City**	Control	Control	Control	Control	Control
**Observations**	1036	1036	1036	1036	1036

Note: Standard errors in parentheses, * p < 0.1, ** p < 0.05, *** p < 0.01.

### Endogeneity test

Endogeneity is primarily induced by two-way causality and omitted variables. To address this issue, this study employed the instrumental variable method, which is based on heteroscedasticity, to construct instrumental variables [87]. The results are shown in Table 6. The p value of the instrumental variable underidentification test is less than 0.001, rejecting the null hypothesis of “insufficient instrumental variable identification” at the 1% significance level. The weak instrument test’s F statistic of 23.38 exceeds the critical value of 21.39 at a 5% bias level, indicating the reliability of the instruments. The results in Table 6 indicate that even after endogeneity is addressed through instrumental variable estimation, the impact of income on farmers’ sense of PSF remains significantly positive. This demonstrates the reliability of the study’s findings.

**Table 6:** Results of instrumental variable regression.

Variable	PSF
**Income**	0.087** (2.03)
**Constant**	3.108*** (19.71)
**Observations**	1036
**R-squared**	0.163
**Underidentification test p-val**	0.0000
**Cragg-Donald Wald F statistic**	23.376

Note: The parentheses show the t statistics. *** p < 0.01, ** p < 0.05, * p < 0.1.

### Robustness check

To rigorously test the robustness of the moderating effect, this study adopts an alternative measurement approach by replacing the originally social dimension-based assessment of Internet embeddedness with an information acquisition dimension. The new measurement items include (1) the frequency of online news browsing, (2) the number of public accounts followed on platforms such as Jinri Toutiao and Weibo, (3) the variety of daily actively followed online information categories, and (4) the quantity of rural service applications routinely used or known.

The factor analysis of selected items yielded satisfactory psychometric properties, as evidenced by the KMO measure of sampling adequacy (0.646 > 0.60 threshold) and Bartlett’s test of sphericity (χ² = 645.890, p < 0.001). These results reject the null hypothesis of variable independence, confirming adequate intercorrelations among questionnaire items for factor analysis. We conducted regression analysis on the data that had been reduced through factor analysis and partitioned into groupings by median and tertiles.

Table 7 presents the results of the Oprobit regression analysis when using an information-based alternative measure of internet embedding, including the results from the median and tertiles quantile groupings. The analysis indicates that with the grouping by median, the moderating effect of internet embedding falls between statistical significance and insignificance. Conversely, with the grouping by tertiles, internet embedding maintains a statistically significant negative moderating effect at the 5% significance level, robustly confirming our main findings. This further underscores the threshold effect of information.

**Table 7:** Oprobit regression results.

VARIABLES	Equation 1	Equation 2	Equation 3	Equation 4
	PSF	PSF	PSF	PSF
**Income**		0.096***	0.212***	0.219***
		(3.25)	(3.22)	(2.66)
**Internet embedding**			0.408***	0.408*
			(3.37)	(1.96)
**Income##Internet embedding (Grouping by tertiles)**			−0.065**	
			(−2.11)	
**Income##Internet embedding (Grouping by the median)**				−0.085
				(−1.59)
**Gender**	−0.101	−0.065	−0.055	−0.063
	(−1.40)	(−0.88)	(−0.75)	(−0.86)
**Age**	0.031	0.027	0.045	0.034
	(0.97)	(0.85)	(1.39)	(1.07)
**Marital status**	0.086	0.068	0.071	0.073
	(1.06)	(0.84)	(0.87)	(0.90)
**Policy identify**	0.188***	0.155**	0.149**	0.165**
	(2.92)	(2.41)	(2.27)	(2.50)
**Education**	0.045	0.027	−0.016	0.011
	(1.48)	(0.85)	(−0.49)	(0.35)
**Length of outside working time**	0.003	0.000	−0.005	−0.002
	(0.13)	(0.02)	(−0.23)	(−0.11)
**Residence time in rural area**	0.068**	0.070**	0.063*	0.069**
	(1.98)	(2.03)	(1.82)	(1.98)
**Diversity of income**	0.054	0.013	−0.014	0.007
	(0.91)	(0.21)	(−0.23)	(0.11)
**Agricultural production**	0.010	0.042	0.060	0.042
	(0.12)	(0.49)	(0.69)	(0.49)
**Distance to town**	0.005***	0.005***	0.004***	0.004***
	(7.46)	(6.44)	(5.50)	(6.13)
**Observations**	1,036	1,036	1,036	1036
**City**	Control	Control	Control	Control

Note: The parentheses show robust z statistics. *** p < 0.01, ** p < 0.05, * p < 0.1.

### Heterogeneity test

The impact of income on PSF may vary among different groups. Hence, this paper analyzes the heterogeneity from the dimensions of “working time” and “living time in rural areas”, and the Oprobit regression results are shown in Table 8. The results of the heterogeneity analysis revealed that the significant results of each group were both significant and nonsignificant and that the heterogeneity was relatively obvious. For farmers who work outside the home for a short period and live in the countryside for a long period, income significantly affects their PSF, and all of these effects are significant at the 1% level.

**Table 8:** Heterogeneity analysis.

VARIABLES	Length of outside working time		Residence time in rural area	
	(1)	(2)	(3)	(4)
	Short	long	short	long
	PSF	PSF	PSF	PSF
**Income**	0.138***	0.042	0.067	0.147***
	(3.42)	(0.95)	(1.28)	(3.96)
**Observations**	534	502	356	680
**Control**	YES	YES	YES	YES

Note: The parentheses show robust z statistics. *** p < 0.01, ** p < 0.05, * p < 0.1.

The results in columns (1) and (2) of Table 8 show that income significantly affects the individual’s PSF for farmers who have worked outside the home for a shorter period, whereas for farmers who have worked outside the home for a longer period, this effect is not significant. The possible reason for this is that farmers who work outside the home for a shorter period earn lower nonfarm income relative to farmers who work for a longer period, and almost all of their income comes from engaging in agricultural production. In addition, as their income increases, they perceive that society is becoming fairer. Conversely, income inequality among farmers in urban areas is relatively high [88], and these farmers face greater life pressures and psychological stress resulting from urban‒rural differences. In such cases, the impact of income increases on enhancing PSF may be insignificant.

The results presented in columns (3) and (4) of Table 8 show that the longer the farmer resided in rural areas within a year, the more significant the effect of income on their sense of fairness, which is significant at the 1% level. However, for farmers who have resided in rural areas for a shorter period within a year, the effect of income on their PSF is not significant. A possible explanation is that the shorter the period of residence in the countryside is, the more materialistic the desire of the farmers whose reference range of income is expanded [18]. In a situation where the average incomes of the reference groups have increased and their household incomes have not widened the gap between them and the average income, the sense of fairness will show a downward trend [89]. Farmers who have lived in the countryside for a shorter period are also likely to be affected by the fact that their incomes have not widened the gap with those of the reference group; thus, their sense of fairness decreases. On the other hand, the Chinese government is currently vigorously promoting rural revitalization, and rural conditions have significantly improved. Farmers who have lived in rural areas for an extended period are more likely to perceive improvements in their living environment. Coupled with an increase in income, they are more likely to perceive increasing PSF.

## Conclusions

How to improve farmers’ social fairness has been a focus of academic attention. Previous studies have focused on the role of income. By integrating social comparison theory and relative deprivation theory, our study provides new insights into this issue by systematically examining how Internet embeddedness alters the relationship between income and PSF through three distinct yet interconnected mechanisms.

This paper utilizes 1,036 research data points from 14 cities/states in Sichuan Province, China, to systematically analyze the relationship between farmers’ income and PSF and the moderating role of Internet embeddedness in the relationship between farmers’ income and PSF by constructing an Oprobit model. In this paper, we verify the stability of the results by changing the moderator variable. This study employs the heteroscedasticity-based instrumental variable method to test for endogeneity. The results show that higher incomes for farmers positively affect PSF and that Internet embedding has a negative moderating effect on the relationship between farmer income and PSF.

First, our findings confirm that income growth remains a fundamental factor in enhancing farmers’ sense of PSF. This result aligns with those of previous studies [55,90] while adding nuance to the ongoing debate about income‒fairness relationships. The positive effect persists even in the digital era, although its magnitude is significantly moderated by Internet usage patterns.

More importantly, we identify three specific mechanisms through which Internet embeddedness weakens this positive relationship: expanded social comparisons and the Internet, which dramatically broadens farmers’ reference groups beyond traditional local comparisons. Where farmers previously compared themselves with 5--10 neighboring households, digital connectivity exposes them to hundreds of potential reference points (Zhu et al., 2020). This expanded comparison scope disproportionately features upward comparisons (Clark & Senik, 2010), as algorithms and human nature both favor attention to more affluent groups. Algorithmic Distortion of Reality: Platform designs systematically amplify the visibility of extreme cases—both positive (e.g., “overnight success” stories) and negative (e.g., “poverty porn”). This creates a distorted representation of the income distribution, where the top 1% receive disproportionate attention (Zhao & Zhang, 2023). Farmers consequently develop skewed perceptions of societal wealth distribution. Cognitive Overload in Digital Environments: The sheer volume of income-related information (estimated at 1,200 + daily exposures) overwhelms farmers’ cognitive processing capacity (Lohmann, 2015). This impairs rational assessment of their relative position, making emotional reactions to salient comparisons more likely.

Our research makes two key theoretical contributions. First, we reconcile conflicting findings about income‒fairness relationships by introducing Internet embeddedness as a crucial moderating variable. Second, we move beyond simplistic positive/negative assessments of Internet effects by specifying the mechanisms through which digital connectivity influences fairness perceptions.

On the basis of our research findings, we believe that to increase farmers’ perceptions of PSF, it is essential not only to increase their income and improve income distribution systems but also to regulate negative online information and strengthen the guidance of public opinion. We suggest that policymakers promote digital literacy programs to help farmers critically evaluate online content and encourage platform designs that present more balanced representations of economic reality.

Future research could explore the following: generational differences in susceptibility to these mechanisms; variations across different types of Internet use (e.g., social media vs. e-commerce); and longitudinal effects as farmers adapt to digital environments. This study highlights the need for a more nuanced understanding of how digital technologies mediate traditional socioeconomic relationships in rural contexts.

## Supporting information

S1 Data Final Supporting Data. (XLSX)
